# Solitary Fibrous Tumors and So-Called Hemangiopericytoma

**DOI:** 10.1155/2012/690251

**Published:** 2012-04-08

**Authors:** Nicolas Penel, Eric Yaovi Amela, Gauthier Decanter, Yves-Marie Robin, Perrine Marec-Berard

**Affiliations:** ^1^General Oncology Department, Centre Oscar Lambret, 3 Rue F Combemale, 59020 Lille, France; ^2^Medical School (EA 2694)—Lille Nord de France University, 59000 Lille, France; ^3^Pathology Departement, Centre Oscar Lambret, 3 Rue F Combemale, 59020 Lille, France; ^4^Oncopediatric Departement, Centre Léon Bérard, 28 Rue Laennec, 69008 Lyon, France

## Abstract

We have reviewed the literature data regarding the spectrum of tumors including solitary fibrous tumor and hemangiopericytoma with special focus on definition of the disease, discussion of the criteria for malignancy, and the key elements of standard treatment of localized disease. We have discussed the emerging concepts on the tumor biology and the different systemic treatments (chemotherapy and molecular-targeted therapies).

## 1. Introduction

Solitary Fibrous Tumor (SFT) constitutes a heterogeneous group of rare spindle-cell tumors that include benign and malignant neoplasms. Their cell of origin is still debated. SFT is preferred by most pathologists as a better term than “hemangiopericytoma” that gathers numerous unrelated entities and is presently only employed by neuropathologists [1]. We focus the present paper on the forms of this family of tumors occurring in adult patients. There are 3 typical primary locations: pleural, meningeal and extrathoracic soft tissue.

## 2. Classical Clinical Forms

### 2.1. Pleural SFT

Most of SFT (80%) are benign neoplasms. They are most commonly diagnosed in adults aged of about 50–70 [2–4]. The M/F gender ratio is about 1. There is no identified risk factor: no association with asbestosis, tobacco consumption or other environmental factor. Pleurodynia, cough, and dyspnea are the most frequent revealing symptoms [2–4]. Most of patients are symptomatic at the time of diagnosis [5]. Two paraneoplastic manifestations could be seen: paraneoplastic osteoarthropathy (pulmonary osteo-arthropathy) and more rarely paraneoplastic hypoglycemia (Doege-Potter syndrome) [4, 6]. In the Magdeleinat series, osteoarthropathy and hypoglycemia occurred in 4/60 and 1/60 cases, respectively [3]. Hypoglycemia is most commonly associated with massive SFT (mean tumor size of about 20 cm), and about 40% of the SFT associated with hypoglycemia are malignant [6]. The mechanism causing this symptom is likely the tumor-producing nonsuppressible insulin-like active substances and insulin-like growth factors (see below). Control of the hypoglycemic episodes occurs rapidly after complete tumor removal.

CT scan classically shows a sessile or a pedunculated pleural mass. Calcifications in the tumor could be seen in both benign and malignant cases [5]. The mean main diameter is about 8 cm (ranged, from 1 to more than 30 cm) [3, 5]. The experience with fluorodeoxyglucose PET is limited; in the Kohler report, PET seemed to be “positive” in 3/3 malignant tumors and negative in 3/3 “benign” SFT [5]. This tumor arises more commonly from the visceral (80%) [3] than the parietal pleura. The vast majority of SFT are covered by a smooth glistering capsule surface [3]. Most of the tumors are composed of uniform collagen-forming spindle-cells, arranged in interlacing fascicles. Some show associated myxoid changes, fibrosis, hyalinization, necrosis, or rarely focal calcifications. The vascularity widely varies. The cells are negative for any anticytokeratin and epithelial membrane antigen, but stain in all cases with antibodies vimentin and CD34 rarely with muscle-specific actin. Because their morphologic pattern is not specific (the so-called patternless pattern) and there are no distinctive immunohistochemical or electron microscopic features, it may be difficult to separate this tumor from other spindle-cell pleural tumors by hematoxylin-eosin staining alone [7].

The criteria for malignancy are presence of necrosis increased mitotic activity (≥4 mitotic figures per 10 HPF), greater cellularity, and cellular polymorphism [4]. SFT could be considered as malignant in the presence of at least one of these criteria [4]. Malignant SFTs tend to be larger and more frequently symptomatic at diagnosis. The presence of associated pleural effusion is more frequent in malignant forms. These criteria for malignancy are still discussed. Two of them are subjective: greater cellularity and cellular polymorphism. About 15 [2, 8] to 35% [3, 4, 9] of SFT are considered malignant at the time of diagnosis and primary treatment. Recurrences are markedly more frequent in malignant cases [5]. The median overall survival of benign forms is about 280 months (5-year OS rate: 80–90%), whereas the median OS of malign forms is about 50–60 months (5-year OS rate: 80–90%) [8].

The cornerstone of the treatment remains the large en-bloc resection, requiring, depending on the local extension, pulmonary wedge resection, lobectomy, pneumonectomy, and sometimes chest wall, diaphragm, or pericardium resection(s) [3, 8]. Mediastinal lymph node dissection is not indicated, as lymph node metastases are rare [3, 8]. Adjuvant treatments (radiotherapy and chemotherapy) are not usually employed [3]. In case of local recurrence, surgery remains the recommended option. In case of distant metastasis or locally advanced recurrence not amenable to surgery palliative radiotherapy and palliative chemotherapy can be considered (see below). There is no consensus on the type of chemotherapy treatment to employ, but, by analogy with other forms of soft tissue sarcoma, doxorubicin ± ifosfamide-based regimen is usually the first choice.

### 2.2. Soft Tissue SFT

Soft tissue SFT is usually diagnosed in the fifth decade. The distribution is equal in both sexes. This tumor represents less than 2% of all soft tissue tumors. Symptoms are not specific. SFT typically appears as a slowly enlarging painless mass. Hypoglycemic episodes have been described, as with SFT in other sites. The tumor is usually located in deep tissue. Cross-sectional imaging (CT-scan and MRI) reveals a heterogenous mass with avid and heterogeneous enhancement after IV contrast administration in most cases [10]. Calcifications were present in fewer than 10% of benign or malignant SFT. Some cases are called lipomatous SFT because some contain fat. There is no distinctive radiological criterion for malignancy, excluding the presence of distant metastases (present at diagnosis in about 10–15% of cases) [10]. The criteria for malignancy remain a topic of some debate. Malignant SFTs are hypercellular and display at least focal moderate-to-severe nuclear atypia. Malignant SFTs often have infiltrative margins with surrounding tissues, have high mitotic count (≥4 mitoses per 10 high-power fields), and display cellular pleomorphism and tumor necrosis. The risk of metastasis is estimated of about 25% [11].

En-bloc surgery is the recommended treatment for both benign and malignant SFT. The risk of peroperative bleeding had to be known. For SFT displaying criteria for malignancy, adjuvant radiotherapy, when feasible is recommended by analogy with other soft tissue sarcomas. There is no evidence supporting the use of adjuvant chemotherapy. Long-term follow is mandatory because of local or distant relapse in malignant and considered benign SFT.

### 2.3. Meningeal Hemangiopericytoma (Solitary Fibrous Tumor)

Meningeal hemangioperictyoma (MHPC) constitutes less than 2% of all meningeal tumors [15]. They have been observed at the falx, occipital and spinal dura, tentorium and cerebellopontine angle. They are first considered as an angioblastic form of meningioma [15]. WHO classification considers MHPC as a member of soft tissue sarcomas of the central nervous system. MHPC is locally aggressive and usually presents as dura-based masses and clinically indistinguishable from to meningiomas. The median age at diagnosis is about 40–50. The clinical and radiological findings are not specific. Surgery remains the cornerstone of the treatment, as other SFT family tumor. Tumor hemorrhage is common during the surgical procedure, and thus the standard of care today often includes preoperative tumor embolisation [16]. Complete resection (R0) is frequently difficult to achieve because these tumors grow along the sinuses. Adjuvant radiotherapy (50–60 Gy) appears to improve the local control rate [17, 18]. Conservative surgery combined with adjuvant radiotherapy has been suggested for tumor with unfavourable location [19]. In the Mena series, local recurrence (2/3 of patients) and extracranial (1/3 of patients) metastases are frequent [20]. Bone, lung, and liver are the most common metastases sites. Metastases could appeared after a long period, usually 5–8 years or more, sometimes more than 20 years [20, 21]. Classical doxorubicin ± ifosfamide-based chemotherapy has been disappointing in the management of metastatic MHPC [18, 22]

### 2.4. Other Forms

SFT could occurred in any site (peritoneal, intrahepatic, intrapulmonary,…). There is no relevant specificity for such locations.

## 3. Focus on Tumor Biology and Systemic Treatment

### 3.1. Classical Chemotherapy

In a retrospective analysis issued in 1978, Wong and Yogda have reported a response of about 50% with doxorubicin-based regimens. This report had to be interpreted with a great deal of caution because (i) the diagnostic criteria were not well established at that time and (ii) the sample size was limited to 16 patients [23]. In a more recent report, Beadle and Hillcoat have reported their experience of 4 patients treated with doxorubicin (50 mg/m² every 4 weeks) and dacarbazine (600–700 mg/m² every 4 weeks). Objective response was seen in 2 cases, stable disease with symptoms relief in one case, and rapid progressive disease in the fourth case [24]. Galanis et al. have found a response rate of 1/7 in patients with meningeal MHPC treated with doxorubicin-based regimens [22]. Park et al. have reported in 2011 at the annual ASCO meetings their retrospective single-center experience (1994–2007) with different chemotherapy regimens: classical doxorubicin-based regimens (*n* = 15), gemcitabine (*n* = 4), and paclitaxel (*n* = 4). The results appeared disappointing with a response rate of 0/15 and a median progression-free survival of about 4 months [25]. Moreover, a case report shows an objective response to trabectedin treatment in heavily pretreated MHPC [26].

### 3.2. Temozolomide and Bevacizumab Combination

Park et al. have reported in 2009 their experience with temozolomide 150 mg/m^2^ orally on days 1–7 and days 15–21 and bevacizumab 5 mg/kg intravenously on days 8 and 22, repeated at 28-day intervals in 14 patients (2005–2007). The median number of cycles of temozolomide and bevacizumab administered was 6.5 (2–20). Grade 3 to 4 toxicities were mainly neutropenia and thrombocytopenia. Best response assessment using so-called Choi criteria showed that 11/14 (79%) achieved a “Choi partial response” (PR), 2/14 (14%) with stable disease (SD), and 1/14 (7%) developed progression of disease (PD). The median progression-free interval was 6 (4–10) months. This report suggested that this regimen could be active and well-tolerated and that a formal phase II trial was needed [27].

### 3.3. IGF2 Pathway

Several case reports demonstrated that hypoglycemia is the consequence of insulin-like growth factor 2 (IGF2) secretion [28–34] or other members of IGF family or IGF protein binding [29]. IGF2 overexpression seems to be the consequence of fetal promoter activation and loss of imprinting in malignant SFT [28]. Hadju et al. have confirmed that IGF2 was uniformly detected in SFT (23/23), regardless of anatomical location and was related to loss of imprinting [35]. Pavelić et al. analyzed the expression of the members of IGF in 7 SFT associated with severe hypoglycemia and 12 cases without hypoglycemia. They found that IGF1 receptor was expressed in 17/19 SFT. Other members of the IGF network are frequently expressed: IGF1 (3/19), IGF2 (11/19), IGF-binding protein 1 (5/19), IGF-binding protein 2 (3/19), and IGF-binding protein 3 (4/19) (Pavelić et al. [36]). Hadju et al. did not find mutation of IGF-R(s) [35]. The *in vitro* inhibition of IGF1 receptor is associated with inhibited proliferation [36]. This IGF network plays a key role in SFT proliferation.

Large en-bloc resection resolves this paraneoplastic syndrome [2–4, 30, 31].

For tumor not amenable to surgery, symptomatic treatment of hypoglycemia requires glucose intake, corticotherapy, or glucagon administration [37]. One case report suggests that radiotherapy could control hypoglycemic episodes in locally advanced disease [38]. Embolisation had been described as a palliative treatment for controlling these manifestations [39]. Despite that SFT are labeled by ^111^ Indium-labelled octreotide scintigraphy, somatostastin failed to ameliorate hypoglycemic episodes [38, 40]. Treatment with somatostatin does not provide response, either [41]. One patient with SFT enrolled in a phase-1 trial exploring the association everolimus-figitumumab experienced partial response [42].

### 3.4. Use of Tyrosine Kinase Inhibitors

Several case reports and retrospective studies suggest that imatinib [43, 44], sorafenib [45], and sunitinib [45, 46] could provide long-lasting stable disease. Tumours responding to imatinib strongly and diffusely expressed PDGFR-alpha, PDGRFR-beta [13, 45]. Rossi et al. described a case with missense mutation on exon 18 of the PDGFR-beta gene [44]. In the Italian experience (10 patients treated with sunitinib), the best response according to the Choi criteria was six partial responses (all with response evaluation criteria in solid tumors stable disease), one stable disease, and three progressive diseases. Evidence of benefit lasted >6 months in five patients [46]. In France, a phase-II trial investigating activity and safety of sorafenib is ongoing. Five patients with progressive disease at study entry have been already enrolled.

## 4. SFT in Children

Paediatric cases of STF are exceptionally reported [13]. The literature review shows only a few paediatric case reports occurring either in Pleura in a 7-year-old boy [13], in Mesentery in a 6-year-old boy [14], or in Orbit [12]. Characteristics of these 3 cases are summarized in Table 1. These 3 tumours presented as nodular, well-encapsulated masses with vascularised component are all diagnosed on morphology and immunohistochemical profile (CD34 positivity) (Table 2).

It is important to first exclude some differential diagnosis among the highly vascular spindle-cell tumours occurring in children: synovial sarcomas, infantile myofibromatosis, and Darrier Ferrand dermatofibrosarcoma (a CD34 positive tumor is also to be excluded in cutaneous or subcutaneous location).

Most often SFTs are regarded as benign but the behaviour is unpredictable only on morphological criteria and there is no strict correlation between morphology and behaviour. Thus long-term followup is mandatory. In the paediatric group, metastatic dissemination is also described [14].

According to the previous reports, outcome depends on the completeness of the tumour resection. Large en-bloc resection and achievement of clear margin reduce the risk of local relapse [4]. SFTs are regarded as chemoresistant tumours [23–27]. But, regarding the lack of data and the rarity of this entity in paediatric populations, the treatment recommendation can only be deduced from adult literature.

## 5. Conclusions

HPC is a controversial entity. SFT could be benign or malignant; criteria for malignancy are discussed. En-bloc resection remains the cornerstone of therapy for curative intent. For tumor displaying evident criteria for malignancy, adjuvant radiotherapy had to be considered. Local and distant relapse can occur more than 20 years after the initial treatment. The activity of classical chemotherapy appears disappointing. Hypoglycemic episodes appear to be explained by activation of  IGFR1, perhaps through excess IGF2 signaling. The IGF1R pathway appears to be an important axis stimulating SFT proliferation. The combination temozolomide-bevacizumab and tyrosine kinase inhibitor acting on VEGF receptors or PDGF receptors warrant formal phase II trials.

## Figures and Tables

**Table 1:** The current partition of the old-term “hemangiopericytoma” (HPC).

True HPC, with myoid/pericytic differentiation	(i) HPC
	(ii) Infantile HPC (infantile myofibromatosis)
	(iii) Glomangiopericytoma/myopericytoma

Solitary fibrous tumour (SFT) group	(i) SFT
	(ii) Giant cell angiofibroma
	(iii) Lipomatous HPC

Other tumours with HPC-like features	(i) Synovial sarcoma with HPC-like features

**Table 2:** Characteristics of the paediatric cases reported in the literature.

Ref.	Location	Age	Size macroscopic aspect	Histology and immunohistochemistry	Outcome
[12]	Mesentery	6 y.o.	10 × 8 × 7 cm Well-defined solid mass Encapsulated tumor Solid and cystic parts	Uniform hypercellular areas of pleomorphic spindle cells intermixed with a dense collagenous stroma with few necrotic areas. 25 mitoses per 50 HPF (i) CD34 positive (ii) NSE positive (iii) DOG-1, Bcl-2, CD117, desmin, S-100 protein, and SMA negative.	No lymph node metastasis was present at diagnosis. Liver metastasis after 8 months

[13]	Pleura	7 y.o.	5 × 5 × 4 cm Well-demarcated nodular mass	Low cellularity (i) CD34, vimentin and bcl2, positive (ii) Cytokeratin, SMA, desmin, and S100 negative	No metastasis. No evidence of tumor recurrence 9 months later

[14]	Orbit	15 y.o.	1.5 × 1.4 × 1.0 cm Well-circumscribed mass Highly vascular	Highly cellular spindle tumor 2 mitoses per 20 HPF (i) AE1/AE2 cytokeratin, smooth-muscle actin, muscle-specific actin, desmine, S100 negative (ii) CD34. and vimentin positive	No metastasis No recurrence after local radiotherapy
